# Comparison of characteristics and immune responses between paired human nasal and bronchial epithelial organoids

**DOI:** 10.1186/s13578-024-01342-1

**Published:** 2025-02-07

**Authors:** Lu Zhu, Wenhao Yang, Jiaxin Luo, Danli Lu, Yanan Hu, Rui Zhang, Yan Li, Li Qiu, Zelian Chen, Lina Chen, Hanmin Liu

**Affiliations:** 1 Department of Pediatric Pulmonology and Immunology, West China Second University Hospital, Sichuan University, Chengdu, China; 2 Key Laboratory of Birth Defects and Related Diseases of Women and Children (Sichuan University), Ministry of Education, Chengdu, China; 3 NHC Key Laboratory of Chronobiology (Sichuan University), Chengdu, China; 4 The Joint Laboratory for Lung Development and Related Diseases of West China Second University Hospital, West China Institute of Women and Children’s Health, West China Second University Hospital, Sichuan University and School of Life Sciences of Fudan University, Sichuan University, Chengdu, China; 5 Sichuan Birth Defects Clinical Research Center, West China Second University Hospital, Sichuan University, Chengdu, China

**Keywords:** Respiratory epithelial cells, Organoids, Immune responses, Respiratory syncytial virus

## Abstract

**Background:**

The nasal epithelium, as part of a continuous and integrated airway epithelium, provides a more accessible sample source than the bronchial epithelium. However, the similarities and differences in gene expression patterns and immune responses between these two sites have not been extensively studied.

**Results:**

Four lines of matched nasal and bronchial airway epithelial cells obtained from the four patients were embedded in Matrigel and cultured in thechemically defined medium to generate patient-derived nasal organoids (NO) and bronchial organoids (BO). Histologic examination of nasal organoid tissue revealed high similarity and a reduced ciliary beat frequency compared to bronchial organoid tissue. Whole exome sequencing revealed that over 99% of single nucleotides were shared between the NO and matched BO and there was a 95% overlap in their RNA transcriptomes. RNA sequencing analysis of differentially expressed genes indicated a significant reduction in the immune response in NO. RSV infection revealed more productive replication in NO, with a downregulated immune pathway identified by RNA sequencing analysis and upregulated levels of pro-inflammatory cytokines in culture supernatants in NO compared to BO.

**Conclusions:**

NO and BO serve as robust in vitro models, faithfully recapitulating the biological characteristics of upper respiratory epithelial cells. The different regions of respiratory epithelial cells exhibit distinct immune responses, underscoring their complementary roles in exploring airway immune mechanisms and disease pathophysiology.

**Supplementary Information:**

The online version contains supplementary material available at 10.1186/s13578-024-01342-1.

## Background

The human airway epithelium, from the nasal cavity to the terminal bronchioles, is covered by the pseudostratified ciliated airway epithelium, which consists of four major cell types: ciliated cells, goblet cells, club cells, and basal cells [1], and play a crucial role in the regulation of immune responses, such as asthma [2]. The development of the bronchoscope has provided physicians and scientists with an ideal tool to obtain bronchial epithelial cells (BEC) for disease diagnosis and basic or translational research. However, this procedure is invasive and carries risks, including bronchial spasm, bleeding and infection [3]. Practical and ethical issues associated with obtaining suitable samples, particularly from children, have hampered the study of BEC in childhood. In contrast, nasal epithelial cells (NEC) are an attractive source of airway epithelial cells because of their accessibility to the tracheobronchial epithelium and the potential for repeated isolation from a single individual. Primary cultures of NEC have been successfully obtained from both nasal biopsies and nasal brushings. Nasal brushing could be a minimally invasive approach to obtaining NEC and is more acceptable for pediatric patients. NEC is technically more readily available than BEC and it is of great interest whether NEC is an ideal surrogate for BEC.

The unified airway theory, which describes the upper and lower airways as a single functional unit [4], has been used to explain inflammatory airway disorders, to better clarify the disease process and to provide a detailed and high-level overview of the dysfunction. Poole et al. [5] observed a 90.2% overlap in expressed genes and a strong correlation in gene expression between the nasal and bronchial transcriptomes. The finding of an overlapping signature in the bronchus and nose supports the potential for biomarker development based on nasal brushes in COPD [6, 7], cystic fibrosis [8, 9], and asthma [10]. NEC and BEC were found to be similar in respiratory syncytial virus (RSV) replication kinetics, cytopathology and innate cytokine responses [11–13]. However, some differences have been reported. For example, Brown et al. reported that immune induction occured more robustly in NEC than in BEC caused by *Haemophilus influenzae* [14], and the same phenomenon appeared to take place in *Bordetella pertussis* [15]. SARS-CoV-2 in NEC and BEC has distinctive transcriptional immune signatures [16], and no common mature miRNAs have been found for NEC and BEC in allergic inflammation [17]. Thus, there is limited or conflicting evidence, and whether NEC could be a surrogate for BEC still seems to be an open question.

Our current understanding of the controversy is limited, in part, by our reliance on studies conducted in model systems that do not fully mimic the in vivo environment. To date, few studies have used paired BEC and NEC to determine the suitability of NEC a a surrogate for BEC, taking into account the influence of genetic factors on cellular responses. In addition, the immortalised nature of cell lines may not accurately reflect their physical and biological properties, such as mucociliary differentiation, gene deficiencies and specific receptors for viral infection, due to the loss of a native cell microenvironment, and could lead to erroneous findings regarding entry, spread and infectivity. Primary epithelial cells cultured via either the 2D method or the air‒liquid interface (ALI) system may proliferate and differentiate in vitro for a finite number of passages before gradually losing their original characteristics. Moreover, the scarcity of human tissue samples obtained from patient biopsies is a significant limitation to in vitro studies using these culture techniques.

Organoids are three-dimensional representations of organs grown from stem cells or patient biopsies that self-assemble over long periods of culture (months) while maintaining genetic and phenotypic stability [18] and providing an almost unlimited supply of primary cells [19]. Recent research has demonstrated the utility of human airway organoids in modelling respiratory diseases, with applications ranging from basic scientific research to pharmaceutical development and clinical diagnosis [11, 20]. However, no publications have characterised or compared the immune responses of paired bronchial (BO) and nasal (NO) organoids.

In the present study, we aimed to obtain paired BO and NO samples from bronchoscopy and nasal swabs from the inferior turbinate of the same patient. We hypothesised that NO could be used as a surrogate marker for BO in studies investigating immune responses.

## Materials and methods

### Patient information and informed consent

The Helsinki Declaration principles were adhered to throughout the study. The cohort comprised four children (two females and two males) aged between 5 and 12 years. The subjects in our study presented with recurrent respiratory infection symptoms suspected to primary ciliary dyskinesia (PCD), requiring bronchoscopy to conducted transmission electron microscopy (TEM) examination and TEM for definitive diagnosis. In the final analysis, PCD was ruled out because of negative TEM and WES examination and normal CBF. PCD was ruled out according to the diagnostic criteria outlined in the European Respiratory Society guidelines [21]. The ethical review board of West China Second University Hospital, Sichuan University (KL118), approved the protocol for the study, and all the family members involved in this study signed informed consent forms in writing.

### Obtaining nasal swabs and bronchoscopy samples

Bronchial tissues were collected via bronchoscopy. A paired inferior turbinate nasal swab sample from the same patient was also obtained via a flocked swab. The residual bronchial tissues and nasal swab samples along the swab head were stored in DMEM separately and transported at 4 °C on ice to the laboratory for further processing within 6 h.

### Establishing child NO and BO

The detailed procedures for bronchoscopy- and nasal swab-derived BO and NO have been described previously by our group [22]. Briefly, the bronchial tissues were subjected to a two-step washing process with cold DPBS (Gibco, c14190500bt) before being immersed in digestion medium containing 400 U/mL collagenase I (Sigma, St. Louis, MO, USA; 9001–12 − 1), 0.25 mg/ml Protease E (Sigma, P5147), 10 µM Y27632 (Selleck, Houston, TX, USA; S6390), and 10 U/ml DNAse I (Sigma, 10104159001) in ADF + + buffer (Advanced DMEM/F12 containing 1× GlutaMAX, 10 mM HEPES, and antibiotics). The tissues were subsequently subjected to agitation at 37 °C for 40–50 min. Following digestion, fetal bovine serum was added to deactivate the collagenase enzyme. The resulting mixture was then filtered through a 40-µm strainer and centrifuged at 200× g for 3 min to isolate the cell pellet.

In accordance with a previously established protocol, slight modifications were made to rinse the nasopharyngeal swab twice with ADF + + buffer to release the majority of the sample into the washing solution and discard the swab. The washing solution was initially filtered through a 70-µm strainer to eliminate paper debris, followed by centrifugation at 200× g for 3 min to retain the cell pellet.

Both of the cell pellets subsequently underwent two washes with ADF + + buffer before being embedded in Matrigel. A suspension containing 30 µL of Matrigel and 5000 cells was then seeded into a single well of a 24-well plate. After solidification at 37 °C for 15 min, 500 µL of previously published airway organoid culture medium was added to each well, and the plates were incubated under standard culture conditions. The airway organoid culture medium included ADF + + buffer, 1× B27 (Gibco, Grand Island, New York, NY, USA, 0080085SA), 5 mM nicotinamide (Sigma, N0636), 1.25 mM N-acetylcysteine (Sigma, A0737), 500 nM SB202190 (Selleck, S1077), 500 nM A-8301 (Selleck, S8301), 500 ng/mL R-spondin1 (R&D, Minneapolis, MN, USA, 4645), 25 ng/mL recombinant human FGF7 (PeproTech, NJ, USA, 450 − 61), 100 ng/mL recombinant human FGF10, 100 ng/mL recombinant human Noggin (R&D, 6057), and 5 µM Y27632 (CST, BOton, MA, USA, 13624), and the culture medium was changed periodically.

The organoids were passaged when they reached a diameter of 100–200 μm. The samples were subsequently resuspended in ADF + + buffer, centrifuged, incubated with TrypLE 1× (Gibco, A1217701), sheared, washed, filtered, and reseeded in Matrigel in a 24-well plate at a density of 5000–6000 cells per well. At each passage, a certain number of organoids were frozen with cryopreservation solution (NCM Biotech, C40100) and stored in liquid nitrogen to meet the requirements of future experiments.

To assess the efficiency of organoid formation, 5000 cells were plated in a 24-well plate and submerged in airway organoid culture medium. The quantification of organoids (defined as structures exceeding 10 μm in diameter capable of further growth) was conducted after 8 days of cultivation via a light microscope (Olympus, Waltham, MA, USA, IX83).

### Whole‑exome sequencing

Exome capture was performed via the xGen Exome Research Panel v1.0 (IDT), followed by paired-end sequencing via the Illumina HiSeq Xten platform (Illumina, Inc., CA, USA). The mean sequencing depth was 278X (ranging from 36 to 576X).

### Read alignment, BAM file generation and post‑alignment optimization

Clean reads were aligned to the reference human genome hg38 (Genome Reference Consortium GRCh38) via the BWA 0.7.17 (Burrows–Wheeler Aligner) MEM algorithm with default parameters. BAM was coordinate-sorted, and PCR duplicates were removed with Sambamba version 0.6.8. After the initial alignment of the WES data, we followed GATK v3.8 Best Practice to process all the BAMs from the same patient. The detailed process was described in our previous studies [23].

### RSV infection

RSV A2 (ATCC, Manassas, VA, USA VR-1540) obtained from Chongqing Medical University was cultured in HEp-2 cells (ATCC), and its viral titer was determined following a standardized protocol [24]. Airway organoids were seeded at a density of 3000 cells per well in a 24-well plate. The organoids were subsequently infected with a solution containing 200 µL of organoid culture medium and 2 µL of virus at a concentration of 1 × 10^8^ pfu/mL in a single 48-well plate. Following a 6-hour incubation at 37 °C and 5% CO2, the infected organoids were washed twice with ADF + + buffer and reseeded as previously described.

### RNA preparation

Total RNA was isolated from the organoids via an RNAprep Pure Micro Kit (Tiangen, Beijing, China, CA, DP420). cDNA was subsequently generated with a Transcriptor First Strand cDNA Synthesis Kit (Roche, Basel, Switzerland, 04897030001) following the manufacturer’s instructions.

### Droplet digital PCR

The droplet digital PCR (ddPCR) procedures adhered to the manufacturer’s guidelines for the QX200 Droplet Digital PCR System utilizing the supermix for probes (no dUTP) from Bio-Rad, Hercules, CA, USA. The final reaction volume was 20 µL and comprised 10 µL of 2× supermix for probes (no dUTP) from Bio-Rad; 2 µL of cDNA derived from 100 ng of RNA from the target sample; 0.5 µL each of the RSV-N-F, RSV-N-R, and RSV-N-P primers; and 6.5 µL of nuclease-free water. The 20-µL mixture was subsequently transformed into droplets via a QX200 droplet generator from Bio-Rad. The droplet-partitioned samples were subsequently transferred to a 96-well plate, sealed, and subjected to cycling in a T100 Thermal Cycler (Bio-Rad) according to the following cycling protocol: an initial step at 95 °C for 10 min for DNA polymerase activation, followed by 40 cycles of denaturation at 94 °C for 30 s and annealing at 58 °C for 1 min, and a final step at 98 °C for 10 min, with a subsequent hold at 4 °C. The cycled 96-well plate was then moved to a QX200 reader (Bio-Rad) for reading in the FAM and HEX channels.

### Histology and immunotaining

The harvested organoids were fixed in 4% paraformaldehyde for 24 h, dehydrated, embedded in paraffin, and serially sectioned at 4 μm. The organoid sections were subsequently subjected to hematoxylin and eosin (H&E) staining. Images were acquired on an Olympus APXVIEW APX100 inverted microscope. The histological diagnosis was determined on the basis of established classification criteria.

For immunofluorescence analysis, the sections were subjected to antigen retrieval by boiling in EDTA solution for 30 min, followed by blocking in 5% BSA buffer for 30 min to minimize nonspecific staining. Primary antibodies targeting ace-tubulin (Santa Cruz, Santa Cruz, CA, USA, sc-23950), P63 (Abcam, CB, Waltham, MA, USA, ab124762), SCGB1A1 (Santa Cruz, Santa Cruz, CA, USA, sc-365992), MUC5AC (Abcam, CB, Waltham, MA, USA, ab198294), and RSV_N () were then applied to the sections, which were subsequently incubated at 4 °C for 24 h. Following washing with PBS for three 10-minute intervals, the sections were subsequently exposed to secondary antibodies labeled with Alexa Fluor 488 (Invitrogen, A11001), Alexa Fluor 594 (Invitrogen, A11012) or Alexa Fluor 647 (Invitrogen, A-21447) for 1 h at room temperature. Nuclei were counterstained with Hoechst 33,342 (Invitrogen, Life Technologies, Carlsbad, CA, USA, H3570). The organoids were subsequently washed three additional times and sealed with anti-fluorescence quenching sealing tablets (YEASEN, Shanghai, China, CA, 36307ES08). Finally, images were captured via a laser-scanning confocal microscope (Olympus, Waltham, MA, USA).

### High-speed microscopy analysis of the ciliary beating frequency (CBF)

The BO and NO were prepared at room temperature (25 °C) for video microscopy with a 40× objective (Sprinter-HD Optronics) to eliminate the influence of environmental factors such as temperature. Three organoid model field images were available for each sample, and movies were recorded at 200 fps to record the ciliary beating frequency (CBF), which was analyzed blindly by two researchers. Three independent tracheal samples and at least 10 cilia in each sample were analyzed. Kymographs of ciliary beating were depicted with a macro embedded in ImageJ [25].

### Cytokine array measurement

The culture supernatants from NO-infected and BO RSV-infected plants were collected after 24 and 48 h and immediately stored at -80 °C. The levels of cytokines in the cell supernatant were measured via a Quantibody Human Inflammatory Array 3 (QAH-INF-3; RayBiotech). Cytokines, including BLC, eotaxin-1, eotaxin-2, GCSF, GM-CSF, I-309, ICAM-1, IFN-gamma, IL-1 alpha, IL-1 beta, IL-1 ra, IL-2, IL-4, IL-5, IL-6, IL-6 R, IL-7, IL-8, IL-10, IL-11, IL-12 p40, IL-12 p70, IL-13, IL-15, IL16, IL-17 A, MCP-1, M-CSF, MIG, MIP-1 alpha, MIP-1 beta, MIP-1 delta, PDGF-BB, RANTES, TIMP-1, TIMP-2, TNF alpha, TNF beta, TNF RI, and TNF RII, are detected. According to the manufacturers’ protocol, the chips were scanned and tested via an Innocan 300 Microarray Scanner.

### RNA sequencing, data processing, and transcriptomic analysis

Standard RNA-seq analyses were performed by Novogene Bioinformatics Institute (Novogene, Beijing, China). The total RNA of each sample was extracted via the TRIzol reagent (Invitrogen), and the integrity of the RNA was assessed via the Bioanalyzer 2100 system (Agilent Technologies, CA, USA). Following the manufacturer’s protocol, next-generation sequencing library preparations were carried out. Libraries with different indices were then multiplexed and loaded on an Illumina instrument, generating 150 bp pair reads. The sequencing method used was “Sequencing by Synthesis”.

The raw data in fastq format were processed via fastp software. An index of the reference genome was created with HISAT2 v2.0.5, and paired-end clean reads were aligned to the reference genome via the same software. FeatureCounts v1.5.0-p3 was utilized to count the reads mapped to each gene, and the FPKM of each gene was calculated on the basis of gene length and read count. Differential expression analysis was conducted with the DESeq2 R package, which employs a model based on the negative binomial distribution. Dispersion and fold change estimates were calculated via data-driven prior distributions. Genes with a Padj value < 0.05 were identified as differentially expressed. The clusterProfiler R package was used for Gene Ontology enrichment analysis, correcting for gene length bias. Enriched pathways with corrected *P* values < 0.05 were considered significant. The Kyoto Encyclopedia of Genes and Genomes (KEGG) database covers genomes, pathways, diseases, drugs, and chemicals (http://www.genome.jp/kegg/). We used the clusterProfiler R package to test the statistical enrichment of DEGs in KEGG pathways.

### Statistical analysis

Each experiment was replicated at least three times. Statistical analyses of the demographic data were performed via Prism (version 8.3.0, GraphPad Software, San Diego, California). To compare the differences between the two groups, paired t tests were used for normally distributed data, whereas Wilcoxon matched-pairs signed rank tests were used for nonnormally distributed data. For comparisons between multiple groups, one-way analysis of variance (ANOVA) followed by Dunnett’s multiple comparisons test was used. The significant differences between the groups are denoted by * *p* < 0.05.

## Results

### Establishment of paired child nasal epithelial organoids (NO) and bronchial epithelial organoids (BO)

**Fig. 1:**
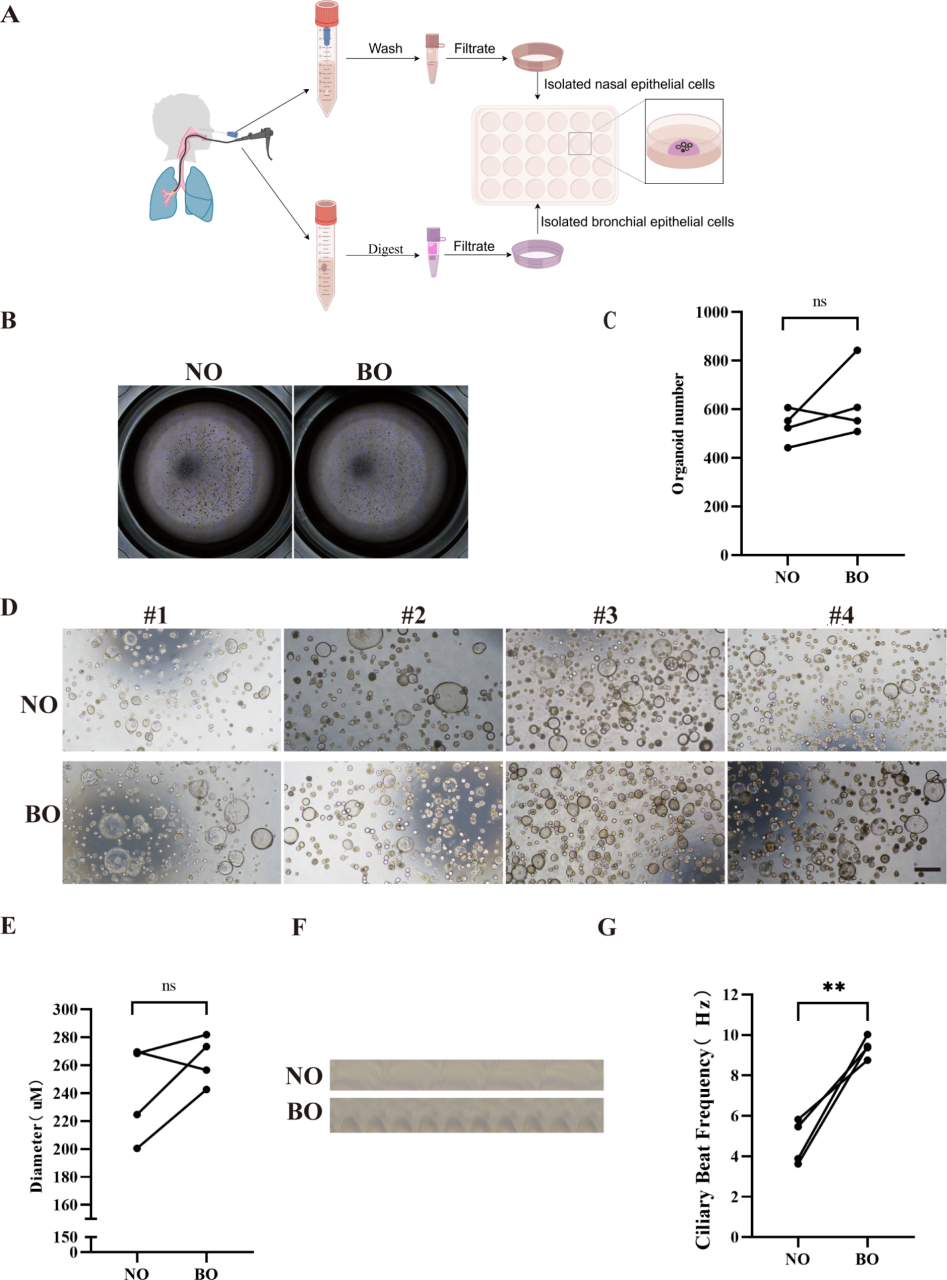
Establishment of child-derived NO and BO. (**A**). Overview of the procedure. The four lines of matched nasal and bronchial tissue models were dissociated into cell clumps and single cells through mechanical disruption and enzymatic digestion. The resulting cells were then embedded in Matrigel and submerged in organoid culture medium. (**B**). Representative organoid images are presented, and the number of organoids was quantified. (**C**). Efficiency of organoid formation in NO and BO. The data presented here represent the mean ± SEM of the number of organoids. (**D**). Representative images of long-term cultured NO and BO are shown, and the organoid sizes were analyzed. Scale bar: 500 μm. (**E**). The organoid size in bright images was measured at D24. The data presented here represent the mean ± SEM of the organoid diameter. ns: not significant; *n* = 20 per group. (**F**). Representative kymograph of ciliary beating in NO and BO. (**G**). The ciliary beat frequency (CBF) in the NO group was significantly lower than that in the BO group (NO vs. BO: 4.70 ± 0.55 Hz vs. 9.40 ± 0.26 Hz, *p* = 0.008). ** *p* < 0.01

Four lines of matched nasal and bronchial tissue models were collected, dissociated into single cells and cell aggregates through mechanical disruption and enzymatic digestion, embedded in Matrigel, and submerged in organoid culture medium to establish primary nasal and bronchial organoids (NO and BO, respectively) [26] (Fig. 1A). There were no significant differences in culture success between the NO and BO samples (Fig. 1B and C), similar to previous reports [20]. Microscopically, highly differentiated NO and BO formed hollow spheres with one or occasionally multiple lumens (Fig. 1D). The growth of these organoids was recorded, and there were no obvious differences in organoid size between NO and BO (Fig. 1E). The ciliary beating frequency (CBF) is a characteristic parameter used to measure the function and integrity of mucociliary defense mechanisms [27]. Ciliary motility was recorded via a microscope equipped with a high-speed digital camera (Additional file 1 and Additional file 2). Kymographs derived from the recordings are shown in Fig. 1F. A comparative analysis of the CBF revealed that NO was significantly lower than that in BO (NO vs. BO: 4.70 ± 0.55 Hz vs. 9.40 ± 0.26 Hz, *p* = 0.008; Fig. 1G). In conclusion, NO and BO retain the in vivo characteristics of children’s airway epithelia and can be indefinitely passaged and frozen for long-term use.

### Immunohistochemical analysis of paired child NO and BO

**Fig. 2:**
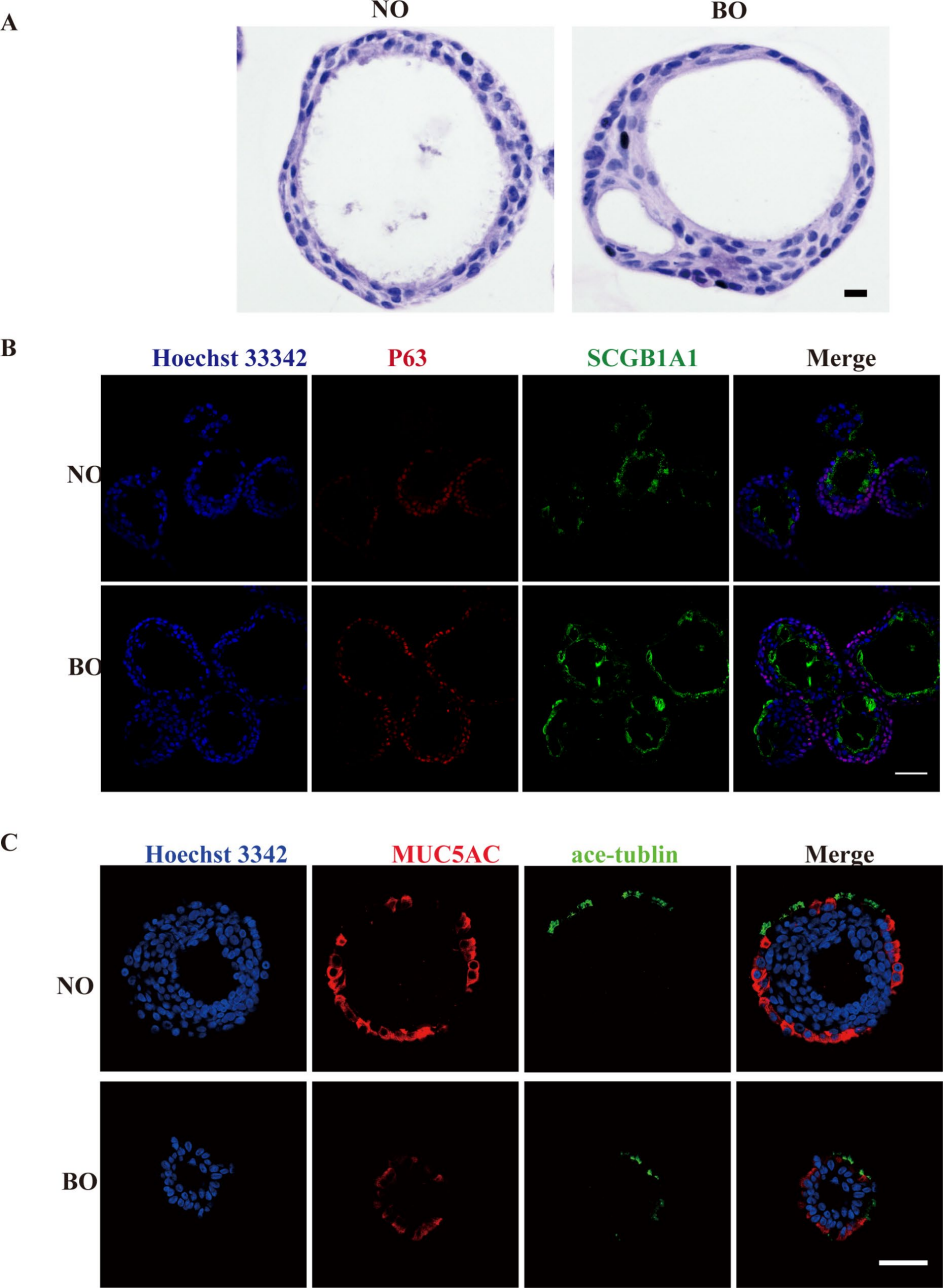
Histological characterization of matched NO and BO. (**A**) Representative images of H&E staining for NO and BO. Scale bar: 10 μm. (**B**, **C**) Representative confocal images showing the expression of respiratory epithelial markers. The confocal images demonstrate the presence and distribution of specific cell types within the organoids, identified by their respective markers: basal cells (P63, red), club cells (SCGB1A1, green), goblet cells (MUC5AC, red), and ciliated cells (ace-tublin, green). Scale bar: 50 μm

To further assess whether the NO resembled the characteristics of their matched BO, we first performed histological analysis. NO displayed analogous histology at the microscale to the corresponding BO, closely mirroring its epithelial organization (Fig. 2A). Consistent with our light microscopy results, these results revealed that the diameters of the organoids were comparable. To gain further insight into the cellular composition and ensure the phenotypic consistency of the organoids, we employed immunofluorescence imaging within the NO and BO. P63 was used to identify basal cells, SCGB1A1 was used to identify club cells, MUC5AC was used to identify goblet cells, and acetylated tubulin (ace-tubulin) was used to identify ciliated cells (Fig. 2B and C). These findings demonstrated that both NO and BO exhibited comparable distributions of these cell types, indicating that the differentiation processes in the organoids were consistent with their respective tissue origins.

### Genetic and gene expression profiles of NO and BO

**Fig. 3:**
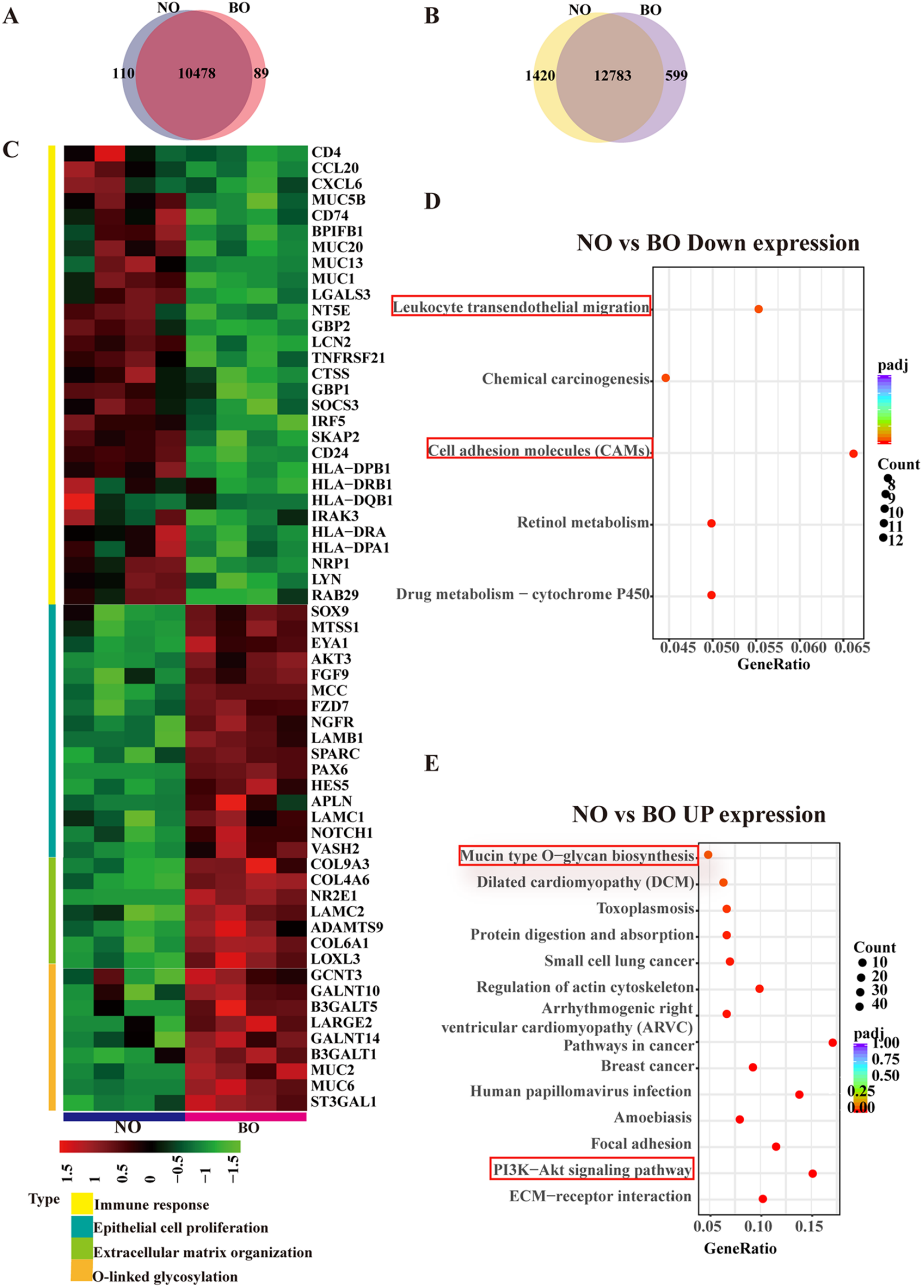
Results of whole-exome sequencing (WES) and RNA sequencing (RNA-seq) in NO and BO. (**A**). Venn diagrams of WES between NO and BO. Venn diagrams depicting the overlap and unique genetic variations identified in the NO and BO through WES. (**B**). Venn diagrams of RNA-seq data between NO and BO. (**C**). Heatmap showing the differential expression profiles of genes between the two study groups and the homogeneity in each group according to RNA-seq. Each row represents a gene, and each column represents an organoid sample. The color gradient indicates the level of gene expression, providing a visual representation of the transcriptional differences. This protein regulates epithelial cell proliferation and protein O-linked glycosylation and adaptive and innate immune response genes between NO and BO. (**D** and **E**) KEGG enrichment analysis of upregulated genes (**E**) and downregulated genes (**D**) in NO compared with BO

To gain further insight into the genetic profiles of NO and BO, we conducted whole-exome sequencing. Our findings revealed that the NO exhibited a highly overlapping (over 99%) single nucleotide variant (SNV) profile with their matched BO, suggesting that the NO recapitulates the genetic information of the BO (Fig. 3A). Subsequently, RNA sequencing (RNA-seq) was performed to ascertain whether NO exhibited a gene expression profile analogous to that of its corresponding BO (Fig. S3). To exclude interindividual heterogeneity with small sequencing samples, we performed principal component analysis (PCA) and correlation analysis on the sequencing data. Correlation analysis revealed notable alterations in gene expression between the NO and BO groups (Fig. S3), whereas intersample differences within the same group were relatively minor (Fig. S3). This finding indicates that biological variations in gene expression data are strongly associated with position effects. The identification of differentially expressed genes (DEGs) was based on a fold change greater than 1 and a *P* value less than 0.05. A total of 12,783 genes were identified as being expressed in both groups, with 95% overlap in expressed genes between the NO and BO transcriptomes. This was accompanied by 1,420 and 599 genes, respectively, that were uniquely expressed in NO and BO (Fig. 3B). The analysis of the sequencing data revealed 2132 upregulated genes and 1036 downregulated genes in NO compared with BO (Fig. 3C and Fig. S3).

On the basis of the expression of canonical markers, we found that the proportions of basal cells (KRT14, KRT4, KRT6A, KRT17, KRT5, KRT7, KRT19, and KRT13), club cells (SCGB1A1, SCGB3A1, S100P, MUC13, MUC1, MUC20, and MUC5B), and ciliated cells (FOXJ1, FOXJ2, HYDIN2, CST1, DNAH1, SNTN, OMG, TPPP3, DNAH5, HYDIN, and ERICH3) were greater in the BO group than in the NO group, whereas the proportion of goblet cells (MUC5AC, MUC2, and MUC6) was greater in the NO group than in the BO group in the control group (Fig. S5), which is consistent with the findings of previous studies.

Compared with BO, the majority of the downregulated genes exhibited either a direct or indirect negative impact on both the adaptive and the innate immune responses in NO. Notable examples include THBS1, PLAUR, IRAK3, HLA-DQB1, CCL20, CXCL6, and CEACAM6. Conversely, the upregulated genes were associated primarily with epithelial cell proliferation and protein O-linked glycosylation processes, encompassing genes such as FGF1, FGF2, FGF4, HES5, GCNT3, and MUC2 (Fig. 3C). To elucidate the functional and biological characteristics of the differentially expressed genes (DEGs), we conducted a Gene Ontology (GO) enrichment analysis focusing on biological processes. In the NO group, genes involved in epithelial cell proliferation, extracellular matrix organization, and growth factor binding were significantly upregulated (Fig. 3D). Conversely, the BO group presented elevated expression of genes associated with immune regulation. Specifically, BO exhibited increased expression in modules involving the interferon-γ-mediated signaling pathway, neutrophil chemotaxis and activation (Fig. 3S). To comprehensively understand the position-related pathways, we performed Kyoto Encyclopedia of Genes and Genomes (KEGG) pathway enrichment analysis, and the results are shown in Fig. 3. Our analysis revealed that mucin-type O-glycan biosynthesis and the PI3K-Akt signaling pathway were upregulated, whereas cell adhesion molecules (CAMs) and leukocyte transendothelial migration were downregulated in NO relative to BO (Fig. 3D and E). These pathways play vital roles in the immune response, indicating that BO may have a heightened capacity for immune surveillance and response to pathogens.

Taken together, these findings highlight the overall conservation of the underlying transcriptional networks between NO and BO. Despite this general similarity, the observed differences in the expression of specific genes were indicative of the function of the specific locus. NO appeared to be more adept at epithelial cell maintenance and differentiation processes, whereas BO was more inclined toward immunomodulatory functions, exhibiting greater immunocytokine regulation and greater immune cell recruitment. This distinction emphasizes the importance of using NO and BO derived from different regions of the respiratory tract to explore the heterogeneity in airway epithelial cell roles and responses. To further explore the role of both proteins in the immune response, we performed viral infection studies using respiratory syncytial virus (RSV).

### Infection of NO and BO with RSV

**Fig. 4:**
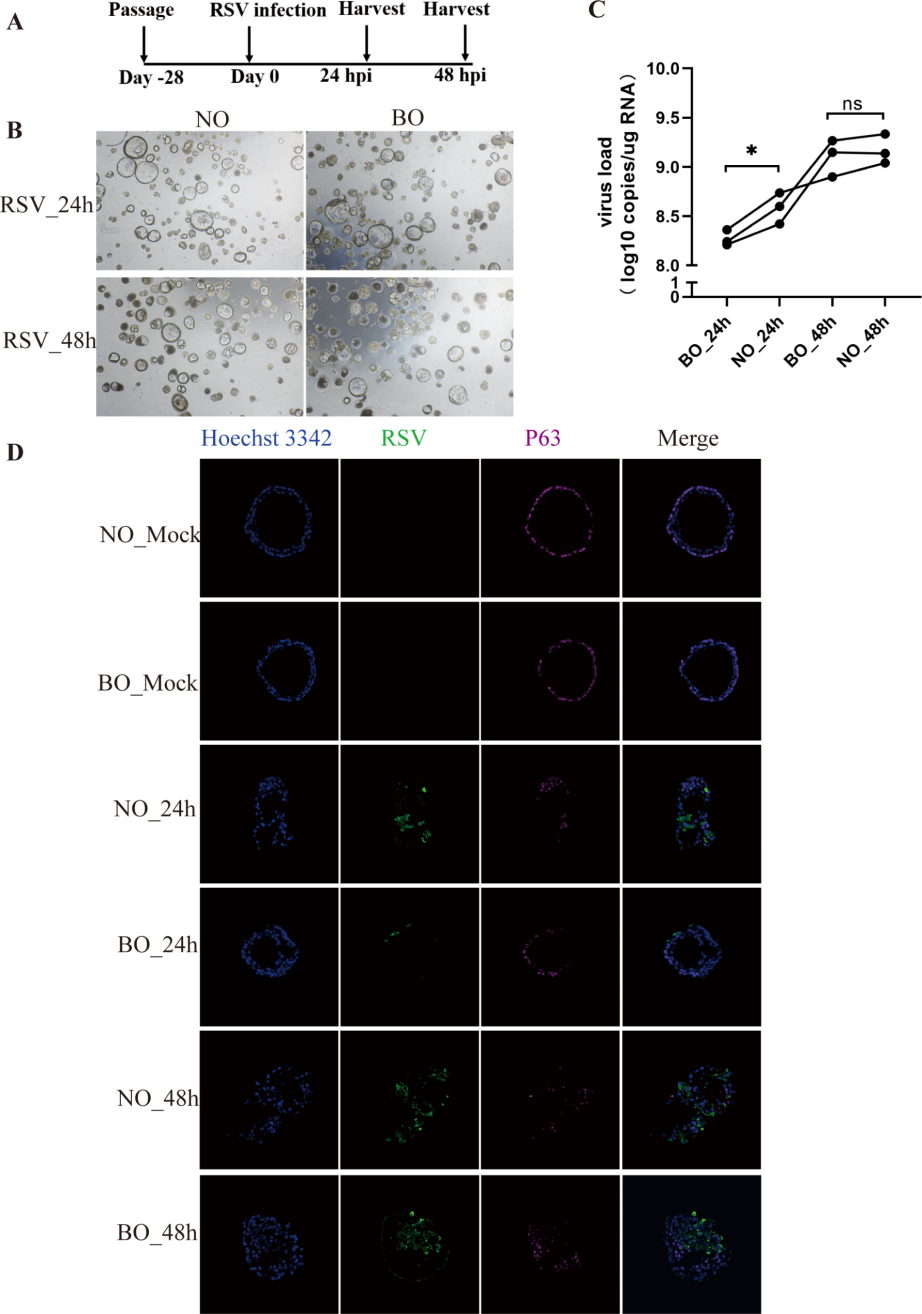
Characterizations of NO and BO after RSV. (**A**). Stepwise protocol for generating organoids infected with RSV. (**B**) Representative light microscopy images of infected NO and BO. Scale bar: 50 μm. (**C**) Quantification of RSV-N gene copies in NO and BO at 24 h post infection (hpi) and 48 hpi. * *p* < 0.05; ns: not significant; *n* = 4 replicates. (**D**) Immunofluorescence analysis of RSV-N protein expression levels. Scale bar: 50 μm

To further elucidate the immune response to an external pathogen, both organoids were subjected to infection with RSV, the predominant virus linked to respiratory infections in infants and young children. Previous research indicated that the peak of RSV replication in NO and BO occurs at 48 h post-infection, which coincides with the initiation of cell death. To investigate the early cellular responses preceding the 48-hour replication peak, NO and BO were infected with RSV for 24 and 48 h post infection (Fig. 4A, and Fig. 4B). The samples were categorized into four groups according to postinfection time points: NO_24 h, NO_48 h, BO_24 h, and BO_48 h. Our findings indicated that the RSV-N gene copy numbers in the NO groups were significantly greater than those in the BO groups following RSV infection, as determined by droplet digital PCR (ddPCR). Furthermore, this difference was statistically significant at 24 h post infection (Fig. 4C). To corroborate our ddPCR results at the protein level, we subsequently employed an immunofluorescence assay to quantify RSV-N protein concentrations (Fig. 4D).

**Fig. 5:**
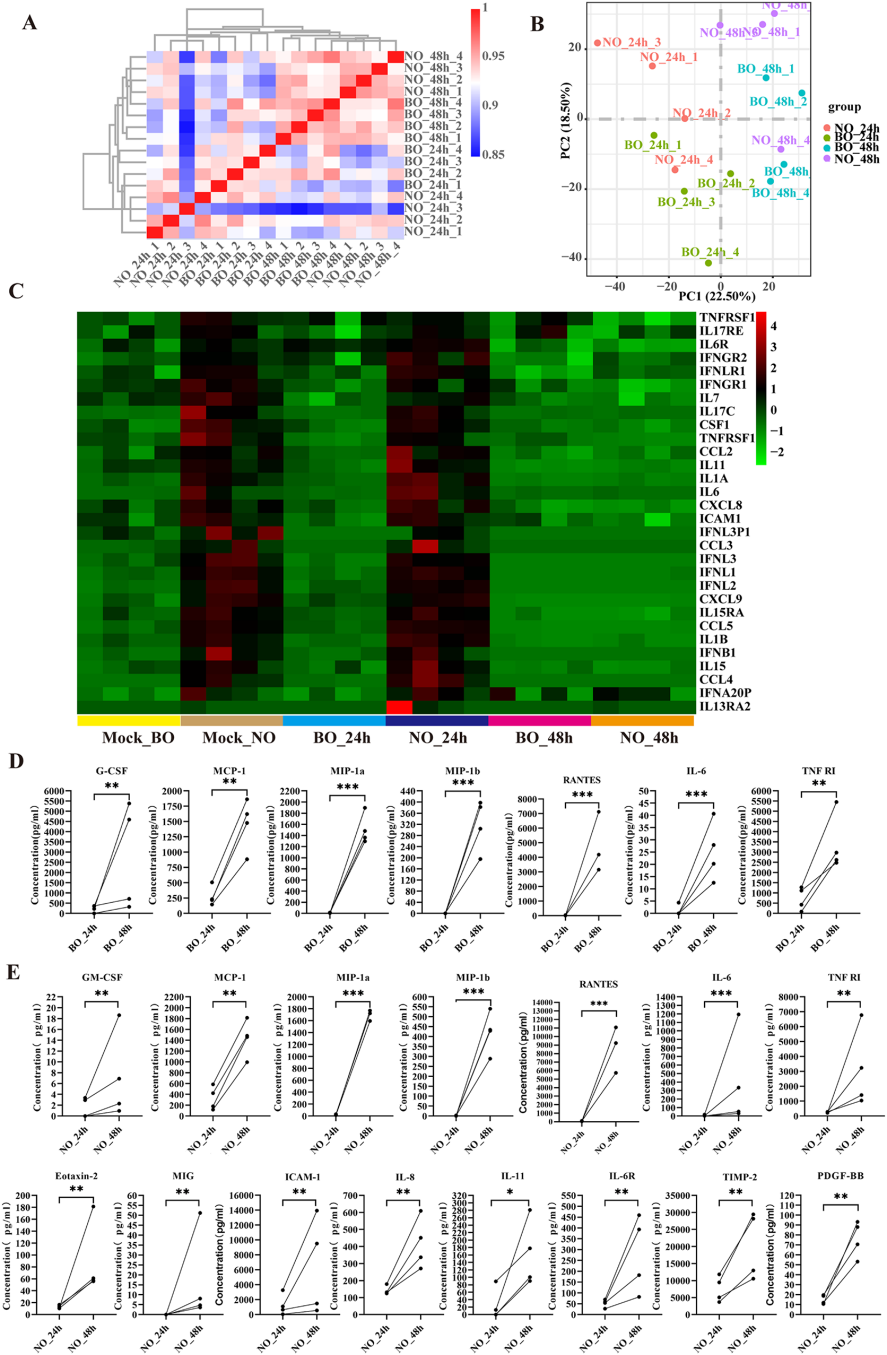
Transcriptomic differences between NO and BO at different stages of RSV infection. (**A**) Pearson correlation heatmap of mRNAs in NO_24h, NO_48h, BO_24h, and BO_48h. *n* = 4. (**B**) Principal component analysis (PCA) of the gene changes. *n* = 4. (**C**) Heatmap of DEGs related to the immune response between groups according to RNA-seq. (**D** and **E**) Expression levels of key immune factors in NO_48h (**E**) and BO_48h (**D**) compared with those in NO_24h and BO-24 h. *n* = 4, ***P < 0.001, **P < 0.01, *P < 0.05

Following infection, total RNA was extracted and analyzed via RNA-Seq. Correlation analyses revealed substantial alterations in gene expression between distinct groups, whereas variations among samples within the same group were relatively minor (Fig. 5A and B). These findings imply that biological variation in gene expression data is significantly influenced by temporal and positional effects.

The analysis revealed that both NO and BO showed an increase in basal cells following infection compared with the control groups. This increase may be linked to the role of basal cells in immune regulation during RSV infection. Moreover, there was a notable increase in the number of secretory epithelial cells, including MUC5AC + goblet cells, in the 24 h and 48 h groups (Fig. S4 and Fig. S5). This finding is consistent with the observed increase in mucus production by the airway epithelium following infection, which suggests an enhanced secretory response to viral challenge.

### RSV infection-driven immune response in NO and BO

We initially examined the distinct inflammatory responses elicited by temporal effects following RSV infection in both NO and BO. DEGs analysis revealed that, compared with those in the mock and 24-hour groups, the NO_48h and BO_48h groups presented elevated levels of inflammation-associated factors. These factors included type I interferons (IFNGR2, IFNGR1, IFNLR1, IFNL3P1, IFNB1, and IFNA20P), interleukins (IL-1B, IL-6, IL-6R, IL-7, IL-11, IL-17RE, and IL-17 C), and chemokines (CCL2, CCL4, CCL5, CXCL8, and CXCL9; Fig. 5C). Notably, compared with those in the BO_24h and NO_24h groups, the levels of genes related to cytokine-cytokine receptor interactions, the TNF signaling pathway, the IL-17 signaling pathway, the chemokine signaling pathway, and the NF-kappa B signaling pathway were increased in the BO_48h and NO_48h groups. These findings suggest enhanced immune activity and response to RSV infection over time (Fig. S5). The observed increased activation of these pathways indicates a more robust and coordinated immune response as the infection progresses.

To evaluate the induction of the acute inflammatory response and the activation of inflammatory cytokines, we quantified their expression levels in culture supernatants via the RayBio Human Cytokine Antibody Array. In NO_48h, the levels of IL-6, MIP-1α, MIP-1β, RANTES, PDGF-BB, IL-6R, IL-8, TIMP-2, MCP-1, eotaxin-2, TNF RI, GM-CSF, ICAM-1, MIG, and IL-11 were significantly elevated compared with those in NO_24h (Fig. 5E). Similarly, in BO_48h, the expression levels of MIP-1α, MIP-1β, RANTES, IL-6, MCP-1, G-CSF, and TNF RI were significantly greater at 48 h than they were in the 24-hour group (Fig. 5D).

### Differences in the immune response between NO and BO

**Fig. 6:**
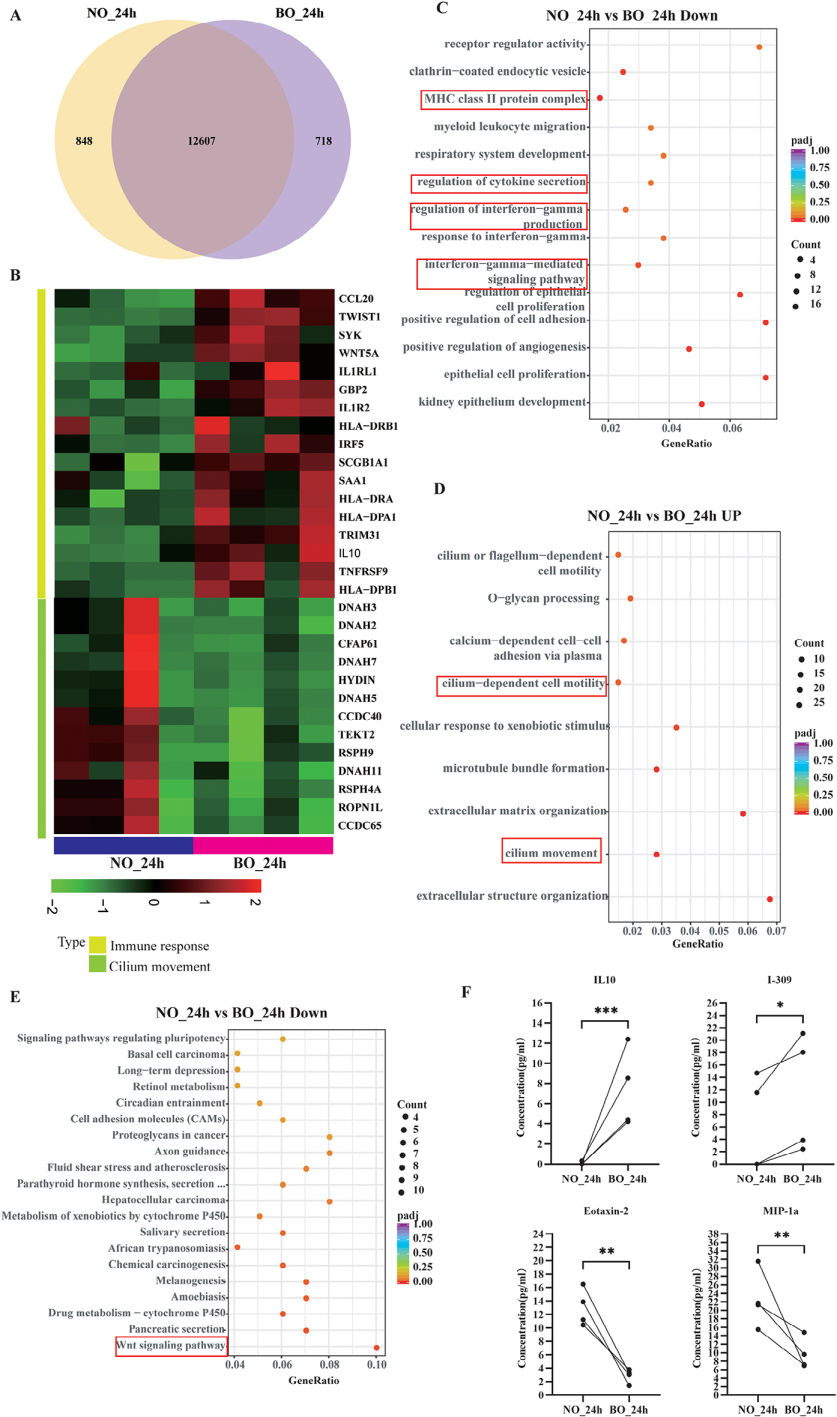
Differences in the immune response between NO and BO after RSV infection at the same time points. (**A**) Venn diagrams of the RNA-seq data showing the number of coexpressed and differentially expressed genes between NO_24h and BO_24h. (**B**) Heatmap illustrating the expression level changes in the statistically significant proteins related to the immune pathway and cilium movement between NO_24h and BO_24h. (**C** and **D**) GO enrichment analysis comparing NO_24h vs. BO_24h. (**E**) KEGG enrichment analysis comparing NO_24h vs. BO_24h. (**F**) Expression levels of key immune factors between NO_24h and BO_24h. (**C** and **D**) GO enrichment analysis comparing NO_48h vs. BO_48h. (**E**) KEGG analysis comparing NO_48h vs. BO_48h. (**F**) Expression levels of key immune factors between NO_48h and BO_48h

To elucidate the similarities and differences in the positional effects of NO and BO immune responses to RSV, we conducted comparative transcriptomic analyses at equivalent postinfection time points. Particular emphasis was placed on the 24-hour postinfection mark, a critical juncture at which NO and BO demonstrate significantly divergent viral copy numbers. We identified 12,607 genes overlapping between NO_24h and BO_24h, 848 genes specifically expressed under NO_24h conditions, and 718 genes specifically expressed in BO_24h (Fig. 6A). Genes involved in ciliary movement, such as DNAH3, DNAH11, HYDIN, CCDC40, DNAH5, and DNAH7, as well as those involved in O-glycan processing, including GALNT14, MUC2, GALNT13, GALNT10, GCNT3, and MUC6, presented increased expression at NO_24h compared with BO_24h (Fig. 6B). Conversely, genes associated with the immune response, such as HLA-DRA, TRIM31, HLA-DPA1, HLA-DRB1, HLA-DPB1, Wnt5a, IL-10, IL1RL1, and HLA-DPB1, were downregulated in NO_24h compared with BO_24h. The GO enrichment analysis of biological processes in NO_24h and BO_24h substantiated the hypothesis that the interferon-mediated immune pathway, cytokine secretion, and epithelial cell proliferation were downregulated in NO_24h relative to BO_24h (Fig. 6C). In contrast, cilium movement and O-glycan processing were upregulated in NO_24h compared with BO_24h (Fig. 6D). While 851 genes were differentially expressed in NO_48h and 489 in BO_48h (Fig. S6), the NO_48h group presented a reduction in the expression of genes associated with epithelial cell proliferation, the transforming growth factor beta receptor signaling pathway, and the ERK1 and ERK2 cascades, as illustrated in Supplementary Fig. 6C. In contrast, compared with the BO_48h group, the NO_48h group presented increased expression of genes related to ciliary motility, as shown in Supplementary Fig. 6D.

Notably, the expression of genes related to cilium movement was greater in the NO groups than in the BO groups at the same time points after RSV infection (Fig. 6G). Our analysis focused on the overall expression levels of genes involved in this pathway and revealed an intriguing trend. In the control group, the expression levels of these genes were lower in NO than in BO, which is consistent with previous analyses of CBF. However, following RSV infection, the expression of cilium-related genes in NO increased significantly at 24 h and 48 h post infection, exceeding the levels observed in BO. However, owing to the isolation requirements for conducting viral experiments, we were unable to obtain CBF after RSV infection. Significantly, KEGG pathway analysis revealed notable upregulation of the Wnt signaling pathway in NO_24h compared with BO_24h, which was increased during infection and inflammation [28] (Fig. S6). This upregulation was driven primarily by the elevated expression of Wnt5a, indicating a distinct difference between NO and BO at this time point (Fig. 6H). Wnt5a is related to the production of anti-inflammatory factors, such as IL-10 and TGF-β, and the suppression of LPS-induced IL-6 and MIP-1a (CCL3) production.

The expression levels of inflammatory cytokines in the culture supernatants demonstrated distinct patterns between the NO and BO groups at the same time points post-RSV infection. Specifically, the levels of IL-10 and I-309 were significantly greater in BO than in NO, whereas eotaxin-2 and MIP-1α were significantly greater in NO at 24 h (Fig. 6F). At 48 h, the expression levels of IL-10 and TNF RII were greater in BO, whereas those of eotaxin-2, IL-6, IL-11, MIG, and RANTES were greater in NO (Fig. S6).

The expression levels of inflammatory cytokines in the culture supernatants demonstrated distinct patterns between the NO and BO groups at identical time points following RSV infection. Specifically, the levels of IL-10 and I-309 were significantly elevated in the BO_24h group compared with those in the NO_24h group. In contrast, the levels of eotaxin-2 and MIP-1α were significantly greater in the NO_24h group than in the BO_24h group (Fig. 6F). At 48 h postinfection, the expression levels of IL-10 and TNF RII were elevated in the BO group, whereas the levels of eotaxin-2, IL-6, IL-11, MIG, and RANTES were increased in the NO group (Fig. S6).

## Discussion

This study demonstrated the establishment and characterization of paired NO and BO derived from nasal and bronchial respiratory epithelial cells of the same child to study host‒pathogen interactions. The histological features of NO and BO were highly similar, and over 99% of single nucleotides were shared between NO and matched BO according to whole-exome sequencing. In contrast to that in BO, a reduction in the ciliary beat frequency according to high-speed microscopy analysis and the immune response according to RNA sequencing analysis were observed in NO. RSV was more replication competent in NO than in BO. The RNA-seq analysis of the samples and the inflammatory cytokine assays of the supernatants revealed that, compared to BO, RSV infection resulted in a significantly reduced immune response in NO, accompanied by an increase in the production of pro-inflammatory cytokines.

The respiratory organoid model, which was first developed in 1993, allows us to efficiently and consistently reconstruct and expand whole human respiratory epithelial cells in a Petri dish. It provides 3D-cultured native human respiratory epithelial cells for routine experiments. Unlike respiratory organoids, NO retains the attributes of the upper airway epithelium and is the first stop for pathogens to enter the respiratory tract anatomically. Earlier studies used invasive nasal brushing samples or biopsy samples, which have traditionally been obtained from human subjects undergoing bronchoscopy procedures [29]. In contrast, we used human nasal swab samples to generate NO in our study, which helped to obtain epithelial samples from the vulnerable pediatric population. Both bronchoscopy and nasal swabs can provide basal cells capable of forming organoids, which recapitulate the cellular diversity of their respective epithelial tissues. However, we acknowledge that bronchial biopsies yield a higher number of basal cells, facilitating faster establishment of organoid cultures. In contrast, nasal swabs yield fewer cells, requiring a longer culture period for sufficient cell expansion. Despite this, nasal swabs offer significant advantages in terms of clinical acceptability and applicability, particularly in pediatric patients and other vulnerable populations, where invasive procedures such as repeated bronchoscopy are less feasible. In addition, to exclude biological and spatiotemporal differences, we collected NEC and BEC from the same individual at the same time point. The significant difference between our study and others is that the respiratory epithelium was strictly pairwise and instrumental. In the current study, our focus is on the use of established in vitro models to characterise heterogeneity and the ability of organoids as in vitro models to mimic altered levels of gene expression in the organism.

The histological features of NO and BO were highly similar and adequately simulated the multicellular composition of the native respiratory epithelium at different anatomical locations and phenocopies of related functions, including ciliary movement (Fig. 2). CBF analysis revealed values within the physiologic range [30], and the BO beat was significantly faster than the matched NO beat (Fig. 1G). Similarly, cilia movement-related genes were found to be transcriptionally expressed at higher levels in BO than in NO (Supplementary Fig. 6). Interestingly, there are some discrepancies between our results and the values reported in previous studies [15]. Some studies have shown no significant differences in CBF when comparing human nasal and bronchial epithelial cell cultures in vitro [31]. However, paired biological samples were used in our study, which provides a more accurate picture of the actual situation. We subsequently investigated the genetic and transcriptomic characteristics of NO and paired BO. The WES technique permitted the examination of the entire coding regions of the genomes, thereby providing a comprehensive overview of the genetic variations present in these organoids. To determine the concordance of the genetic information of NO and BO, we performed single nucleotide variant (SNV) comparisons of NO and its matched BO. Comprehensive genetic analysis revealed that the SNV traits of NO and its matched BO highly overlapped (more than 99%) (Fig. 3A). This high degree of genetic concordance suggests that NO may be a viable alternative to BO in the study of other genetic mutation diseases, such as primary ciliary dyskinesia [22], cystic fibrosis [32] and COPD [6]. RNA-seq analysis revealed that 12,783 genes were expressed in both NO and BO, with 95% overlap between their transcriptomes, indicating a high degree of similarity in gene expression profiles between the two types of organoids. Similarly, Kicic et al. [33] revealed that approximately 91% of the expressed genes were homologous between the upper and lower airways of children via RNA sequencing of epithelial cells from two sites via differential gene expression and gene coexpression analyses to assess transcriptional similarity. Roberts et al. [34] concluded that nasal brushings could be surrogates for bronchial brushings by defining the role of the type 2 cytokine IL-13 in regulating proinflammatory and anti-rhinovirus responses within nasal and bronchial airway epithelial cells taken from the same patient. Furthermore, studies demonstrated that NECs may serve as a suitable alternative to BECs for studying RSV human airway epithelial cell interactions, as many hallmarks of RSV cytopathogenesis are evident in these models [12, 13, 35]. Thus, studying NEC may potentially provide some insights into the entire respiratory epithelium.

Human RSV causes bronchiolitis and pneumonia in infants and young children globally, leading to extensive morbidity and nearly 200,000 deaths per year [11]. We are the first to show the differential susceptibility of immune responses of NO and paired BO to RSV. The results demonstrated that the number of RSV-N gene copies in NO was greater than that in BO following RSV infection, as determined by ddPCR (Fig. 4C). Interestingly, this result contrasts with the results of studies on RSV infection, which previously showed that BECs are more capable of viral replication [12, 13]. Notably, at the transcriptomic level, prior to RSV infection, we observed higher expression of genes related to immune regulation in BO compared to NO. Specifically, BO_24h showed elevated expression in modules associated with interferon-γ-mediated signaling pathways, neutrophil chemotaxis, and activation compared to NO_24h. In addition, the expression of genes related to cilium movement was greater in the NO groups than in the BO groups at the same time points after RSV infection (Supplementary Fig. 6). This shift in gene expression indicates a dynamic response of the nasal epithelium to RSV infection. The elevated expression of cilium movement genes in NO postinfection may indicate an intensified effort by the nasal epithelium to maintain or restore ciliary function, which is essential for the clearance of pathogens and mucus from the airway. Furthermore, this elevated gene expression is consistent with the fact that the nasal epithelium is often the initial site of RSV infection and meets the virus before it reaches the bronchial epithelium. Thus, early robust activation of cilia-related genes in NO may be part of an initial defense mechanism that primarily mitigates the effects of infection and maintains airway patency.

The host’s innate immune response may influence early respiratory transmission. To further investigate the difference in the immune response of the organs of the two species after viral infection, we collected their postinfection culture supernatants for cytokine assays. The results revealed that the two types of organs presented different cytokine responses after infection. Specifically, eotaxin-2 and MIP-1a were more highly expressed in NO_24h than in BO_24h, and eotaxin-2, RANTES and MIG were more highly expressed in NO_48h than in BO_48h. Eotaxin-2, MIP-1a [36], RANTES [37] and MIG [38] belong to a family of chemokines that govern the earliest stages of the inflammatory response, leading to the release of inflammatory mediators by eosinophils, the promotion of Th2 inflammatory responses, and the regulation of IgE synthesis [39]. Eosinophilic inflammation is a characteristic feature of RSV-related asthma [40]. In addition, eotaxin-2 plays a role in both small and main airway obstruction [41] and is responsible for MUC5AC overexpression in airway inflammation by activating ERK1/2 and p38 MAPK [42], leading to mucus metaplasia. The increased expression of MIP-1α has been associated with the severity of illness and a longer duration of supplemental oxygen therapy in children with RSV-related lower respiratory tract infection [43]. Studies have demonstrated that increased RANTES levels in nasal epithelia at the time of bronchiolitis are associated with an increased likelihood of subsequent asthma [44] and greater airway resistance and methacholine hyperresponsiveness [45]. Interestingly, MIP-1α, eotaxin, and RANTES are also associated with the proliferation and survival of airway smooth muscle, leading to airway tissue remodeling through the activation of p42/p44 MAPK [46]. These chemokines are of interest in the pathogenesis of RSV and align with our finding that eotaxin-2, MIP-1a, RANTES, and MIG are highly expressed in NO compared with BO post-RSV, possibly indicating the severity of inflammation and poor disease prognosis in NO.

Classically, IL-6 is known to be a key mediator of inflammatory disease, is involved in promoting the differentiation of naïve CD4 + and CD8 + T cells and is an important link between innate and acquired immunity [47]. High IL-6 levels are correlated with the need for ventilation and with a high degree of hypoxia [48] and could be reliable biomarkers for determining the severity of RSV infection [49]. IL-11 is a member of the IL-6 family, and both cytokines form a similarly arranged gp130 heterodimer complex to initiate downstream signaling [50]. Here, we reported that the expression levels of IL-6 and IL-11 were greater at NO_48h than at BO_48h. At this time point, the greater copy number of RSV in the NO group than in the BO group was accompanied by elevated IL-6 and IL-10, further confirming the differential expression of immune responses in the BO and NO groups.

Interestingly, NO elicited a more profound anti-inflammatory cytokine response characterized by increased levels of I-309, TNF RII and IL-10 than did BO post-RSV. I-309 acts as a mediator of macrophage M2 polarization [51]. The increased expression of I-309 was associated with fewer relapses in RSV-induced first wheezing episodes [52]. TNF RII is a TNFR superfamily molecule that plays an obligatory role in immune homeostasis by suppressing excessive immune responses [53]. It also elicits strong anti-inflammatory activities and has protective effects on cardiomyocytes [54] and Tregs [55]. IL-10 triggers the conversion of M1 macrophages into M2 macrophages [56] and maintains the epithelial barrier and secretory function [57]. Data from clinical studies have shown that increased levels of IL-10 are associated with disease remission [58]. These findings are in line with our results that both BO_24h and BO_48h elicited a more profound anti-inflammatory cytokine response characterized by increased levels of IL-10, I-309, and TNF RII and decreased levels of proinflammatory factors, chemokines, than NO_24h and NO_48h did, which revealed that RSV-N gene copy numbers were greater in the NO group than in the BO group at the corresponding time points.

The Wnt signaling pathway is involved in a variety of life processes, and Wnt5a expression is increased during infection and inflammation, which can regulate the production of the immune anti-inflammatory factor IL-10^28^. Notably, Wnt5a levels were increased in both the BO_24h and BO_48h groups compared with those in the NO_24h and NO_48h groups in our study, accompanied by elevated IL-10 expression in the culture supernatant, which may explain the differences in inflammatory responses between the groups. Oderup et al. reported that recombinant Wnt5a stimulated the secretion of IL-10 through noncanonical signaling cascades, including PI3K (PTEN and AKT), MAPK, and NF-kB signaling components; promoted Treg generation; suppressed the LPS-induced production of the proinflammatory cytokines IL-6 and MIP-1a by normal lymph node DCs; and reduced TNF-a [59]. In line with this report, Valencia et al. reported that Wnt5a-induced production of IL-10 was not a consequence of β-catenin accumulation but was dependent on noncanonical Ca2+/calmodulin-dependent protein kinase II/NF-kB signaling [60]. In contrast, Yaguchi et al. [61] found that IL-10 expression was associated with β-catenin accumulation in human melanoma cell lines and tissues and was induced by direct β-catenin/TCF binding to the IL-10 promoter. Cheng et al. [28] also reported that Wnt5a induced IL-10 secretion in septic mice, which was accompanied by activation of the Wnt/β-catenin signaling pathway, Treg induction and regDC differentiation. In line with our results, an increase in Wnt5a was accompanied by an increase in IL-10 and proinflammatory cytokines, which may have contributed to the lower RSV-N gene copy numbers in the BO group. Whether Wnt5a-induced IL-10 elevation occurs via PI3K (PTEN and AKT), MAPK and NF-kB, or b-catenin in our model remains to be further investigated. If confirmed, this study provides new strategies and targets for the treatment of RSV and other infection-related diseases.

## Conclusion

In summary, we established and characterized NO and paired BO to study host‒pathogen interactions, reflecting the structural and functional organization of the respiratory epithelium in children. Our findings demonstrated similarities in morphology and single nucleotide variant profiles between NO and paired BO. RSV infection of NO and BO resulted in more productive viral replication in NO than in BO, alongside differential immune responses, including downregulated immune pathways and elevated levels of proinflammatory cytokines in NO compared with BO. These findings underscore that the different regions of respiratory epithelial cells exhibit distinct immune responses, highlighting the complementary roles of NO and BO in exploring airway immune mechanisms and disease pathophysiology. This positions NO and BO as valuable models for advancing our understanding of respiratory diseases and host-pathogen interactions.

## Electronic supplementary material

Below is the link to the electronic supplementary material.

**Additional file 1**: Video of ciliary motility in BO were recorded via a microscope equipped with a high-speed digital camera.

**Additional file 2**: Video of ciliary motility in NO were recorded via a microscope equipped with a high-speed digital camera.

**Additional file 3**: **Fig. S3** Bulk RNA sequencing (RNA-seq) of four lines of NO and BO. A Pearson correlation heatmap of mRNAs in the NO and BO groups (*n* = 4). B PCA of the gene changes. C Volcano plot of DEGs. The green and red dots in the plot represent the DEGs with statistical significance. Red represents the upregulated genes, green represents the downregulated genes, and blue represents the genes whose expression did not significantly change. D and E. GO enrichment analysis of upregulated genes (D) and downregulated genes (E) in NO compared with BO.

**Additional file 4**: **Fig. S4** MUC5AC + goblet cells in the 24 h and 48 h groups.

**Additional file 5**: **Fig. S5** Transcriptomic differences between NO and BO at different stages of RSV infection. A. Venn diagram showing the overlap of differentially regulated genes identified from NO_Mock, NO_24h and NO_48h. B Venn diagram showing the overlap of differentially regulated genes identified from BO_Mock, BO_24h and BO_48h. C Heatmap illustrating the differential expression of four epithelial cell marker genes, as well as the degree of homogeneity, between the study groups by RNA-seq. D KEGG enrichment analysis of upregulated genes in NO_48h (D) and BO_48h (E) compared with NO_24h and BO-24 h.

**Additional file 6**: **Fig. S6** Differences in the immune response between NO and BO after RSV infection at the same time points. Venn diagrams of the RNA-seq data showing the number of coexpressed and differentially expressed genes between NO_48h and BO_48h. B Heatmap illustrating the expression level changes in the statistically significant proteins related to the immune pathway and cilium movement between NO_48h and BO_48h.

## Abbreviations

NEC: Nasal epithelial cells
BEC: Bronchial epithelial cells
NO: Nasal organoids
BO: Bronchial organoids
RSV: Respiratory syncytial virus
