# Supplementary figures and images for: Comparison of characteristics and immune responses between paired human nasal and bronchial epithelial organoids
Source: Cell Biosci. 2025 Feb 7;15:18. doi: 10.1186/s13578-024-01342-1 (PMC11806626; doi:10.1186/s13578-024-01342-1)

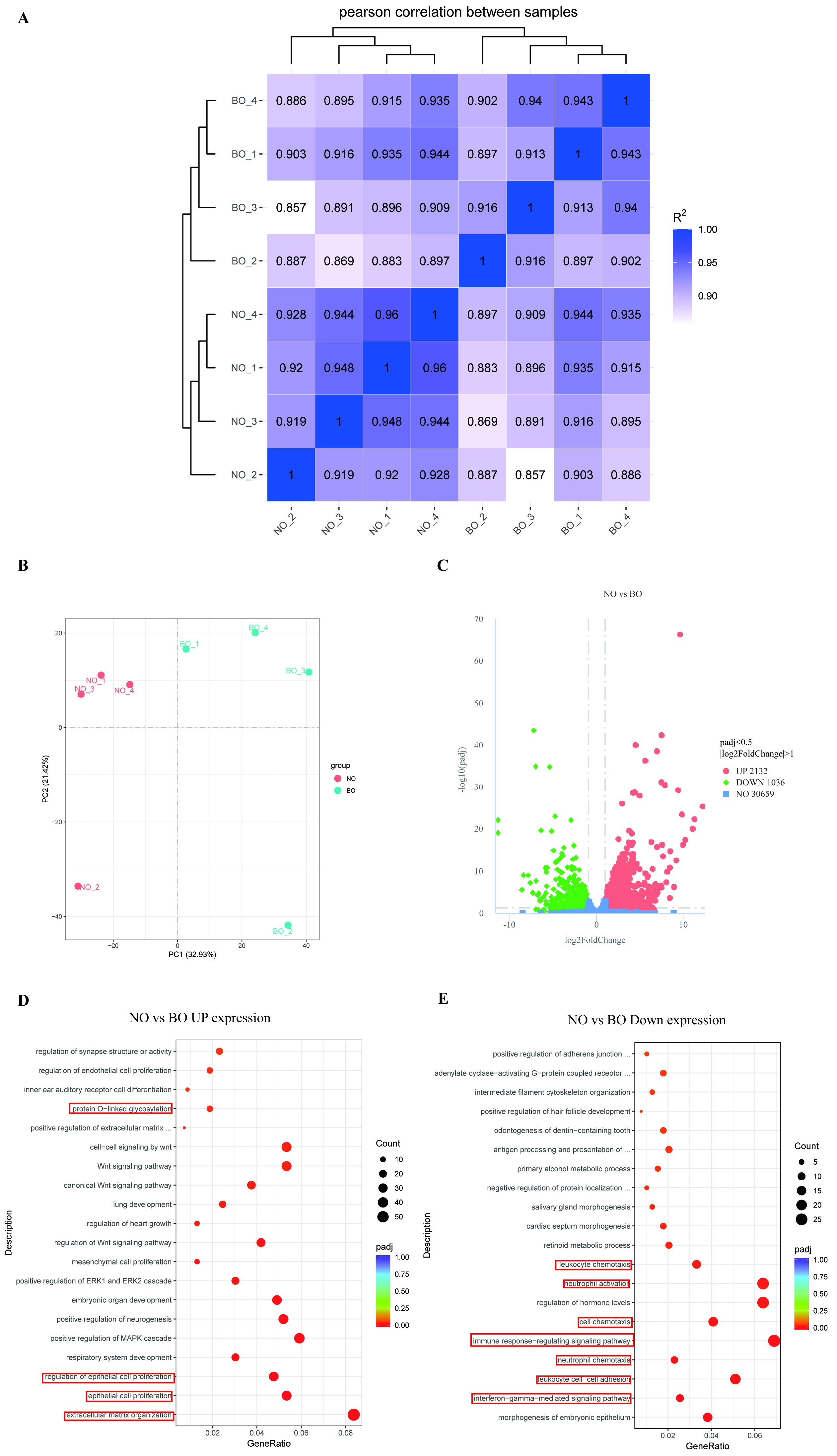

Supplement: Supplementary file 3 — Additional file 3: Fig. S3 Bulk RNA sequencing (RNA-seq) of four lines of NO and BO. A Pearson correlation heatmap of mRNAs in the NO and BO groups (n = 4). B PCA of the gene changes. C Volcano plot of DEGs. The green and red dots in the plot represent the DEGs with statistical significance. Red represents the upregulated genes, green represents the downregulated genes, and blue represents the genes whose expression did not significantly change. D and E. GO enrichment analysis of upregulated genes (D) and downregulated genes (E) in NO compared with BO. [file 13578_2024_1342_MOESM3_ESM.tif]

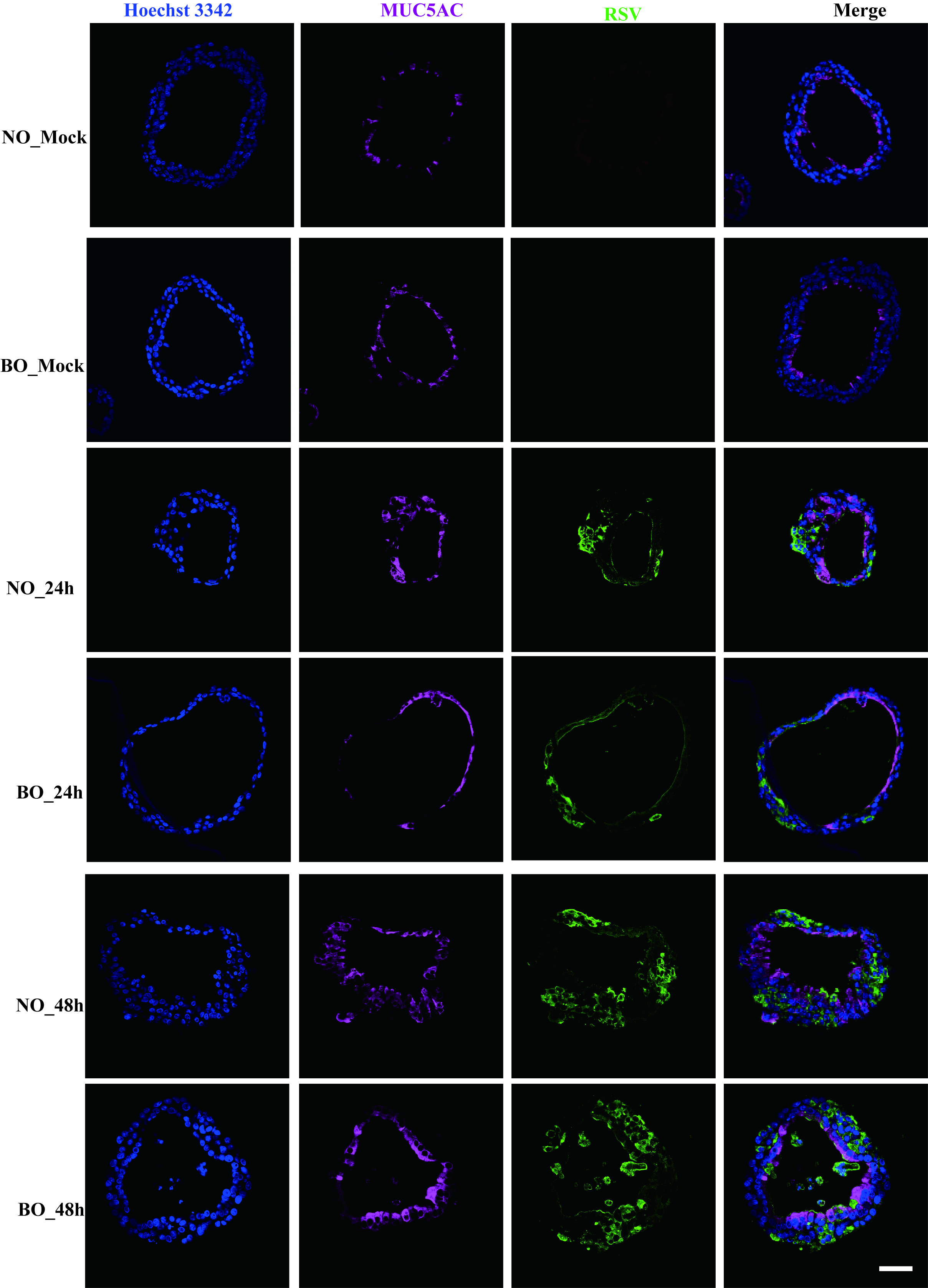

Supplement: Supplementary file 4 — Additional file 4: Fig. S4 MUC5AC + goblet cells in the 24 h and 48 h groups. [file 13578_2024_1342_MOESM4_ESM.tif]

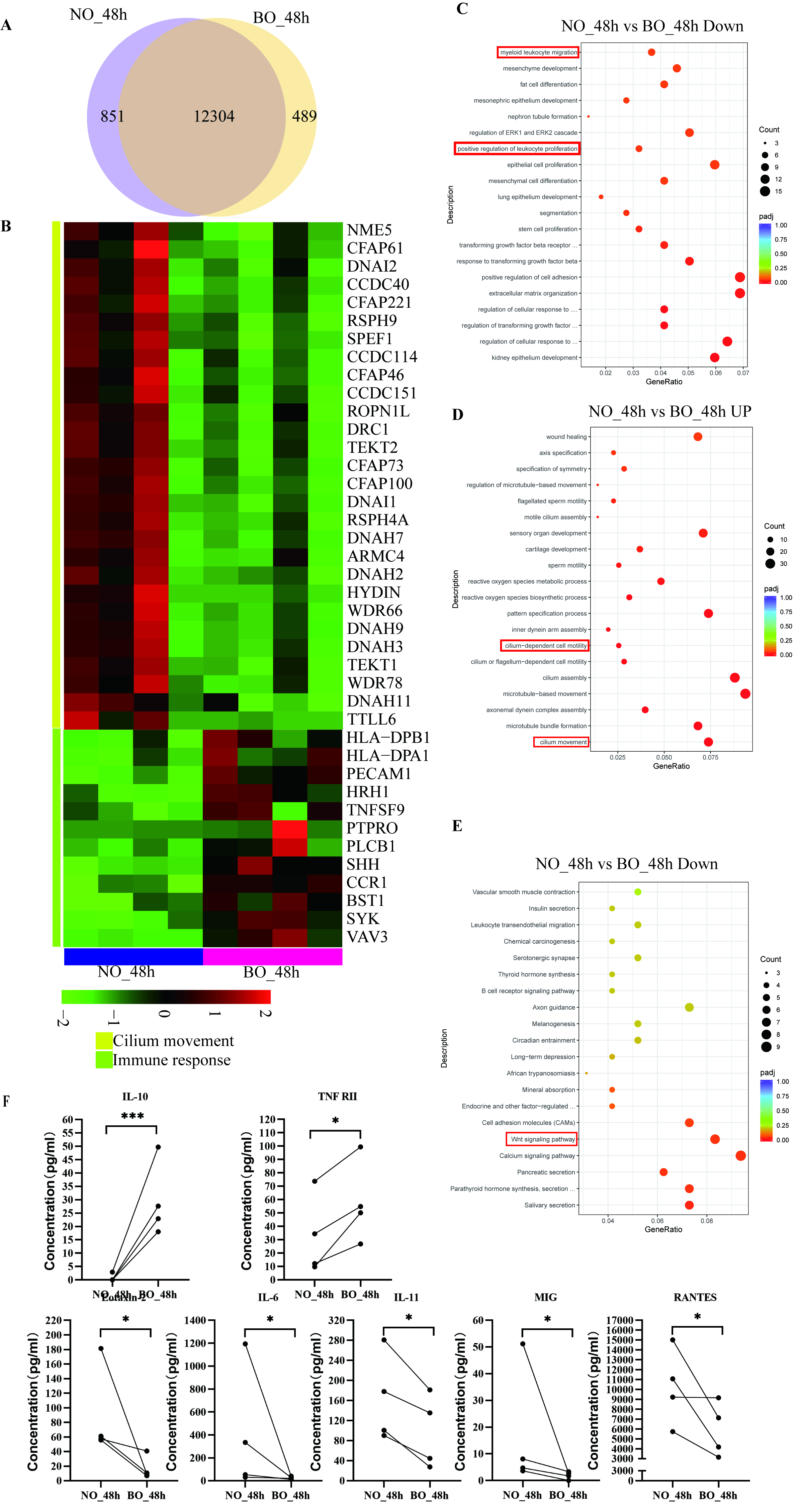

Supplement: Supplementary file 5 — Additional file 5: Fig. S5 Transcriptomic differences between NO and BO at different stages of RSV infection. A. Venn diagram showing the overlap of differentially regulated genes identified from NO_Mock, NO_24h and NO_48h. B Venn diagram showing the overlap of differentially regulated genes identified from BO_Mock, BO_24h and BO_48h. C Heatmap illustrating the differential expression of four epithelial cell marker genes, as well as the degree of homogeneity, between the study groups by RNA-seq. D KEGG enrichment analysis of upregulated genes in NO_48h (D) and BO_48h (E) compared with NO_24h and BO-24 h. [file 13578_2024_1342_MOESM5_ESM.tif]

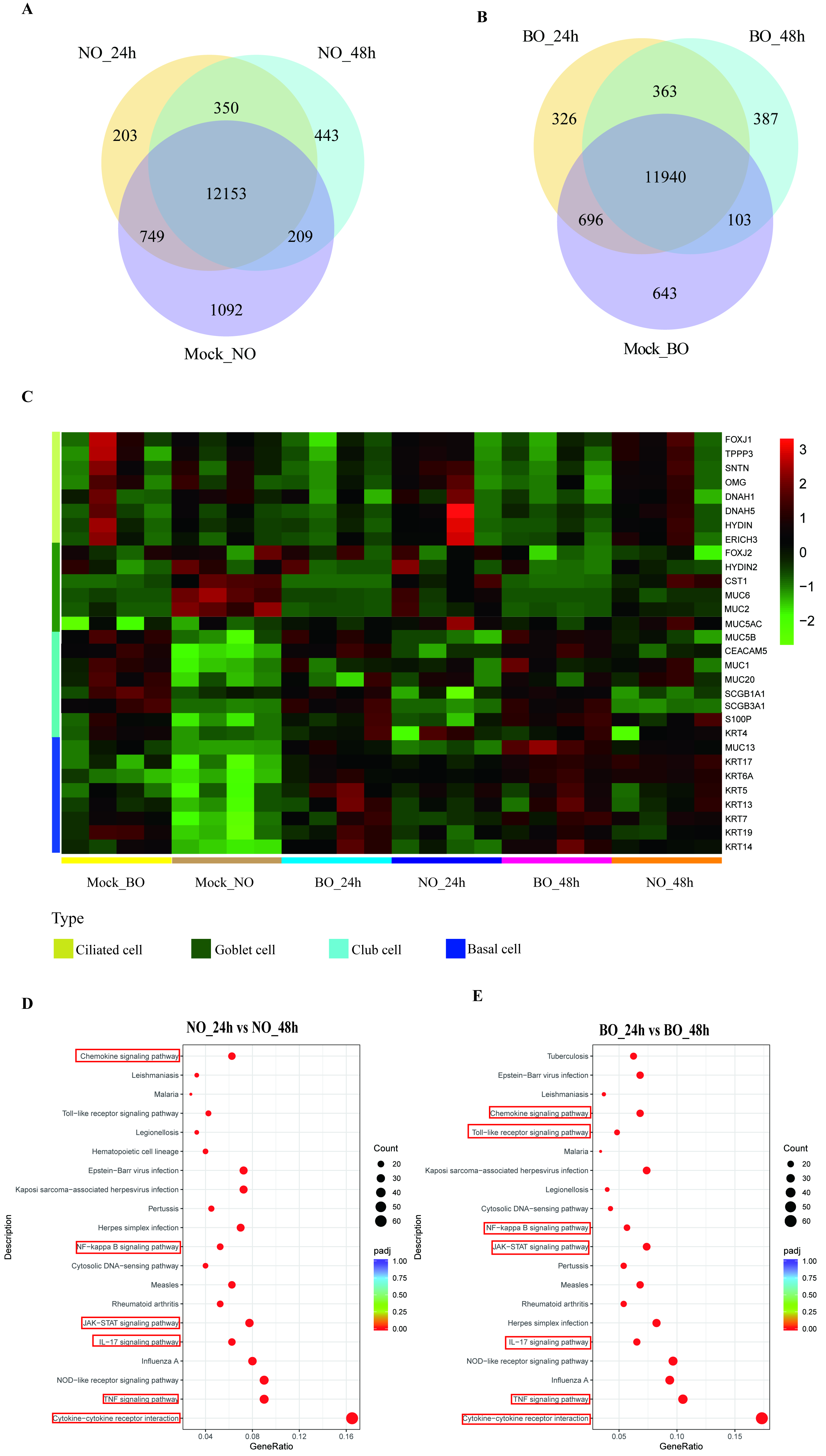

Supplement: Supplementary file 6 — Additional file 6: Fig. S6 Differences in the immune response between NO and BO after RSV infection at the same time points. Venn diagrams of the RNA-seq data showing the number of coexpressed and differentially expressed genes between NO_48h and BO_48h. B Heatmap illustrating the expression level changes in the statistically significant proteins related to the immune pathway and cilium movement between NO_48h and BO_48h. [file 13578_2024_1342_MOESM6_ESM.tif]
